# Emergency repair of proximal hard-tube connector crack during veno-arterial extracorporeal membrane oxygenation support: a case report

**DOI:** 10.3389/fcvm.2026.1707214

**Published:** 2026-03-05

**Authors:** Fengyun You, Yanping Wei, Jing Zheng, Xiu Jiang, Ying Lin, Meizhen Zou, Bin Lin, Yang Lin, Jun Ke, Jianghu Chen

**Affiliations:** 1 Fujian Provincial Key Laboratory of Emergency Medicine, Department of Emergency, Fujian Provincial Hospital, Fuzhou University Affiliated Provincial Hospital, Shengli Clinical Medical College of Fujian Medical University, Fuzhou, China; 2 Nursing Department of School and Hospital of Stomatology, The School of Nursing, Fujian Medical University, Fuzhou, China; 3 Department of Nursing, Fuzhou University Affiliated Provincial Hospital, Shengli Clinical Medical College of Fujian Medical University, Fuzhou, China; 4 The Third Operating Room, Fuzhou University Affiliated Provincial Hospital, Fuzhou, China; 5 Center for Information Management, Fuzhou University Provincial Affiliated Hospital, Fuzhou, China; 6 Fuzhou University Affiliated Provincial Hospital, Fuzhou, China; 7 Department of Anesthesiology, Fuzhou University Affiliated Provincial Hospital, Fujian Provincial Hospital, Shengli Clinical Medical College of Fujian Medical University, Fuzhou, China; 8 Emergency Medicine, Fujian Provincial Key Laboratory of Critical Care Medicine, Fujian Provincial Co-Constructed Laboratory of “Belt and Road”, Fuzhou, China

**Keywords:** bone wax repair, emergency repair, hard tube connector crack, low-flow operation, V-A ECMO

## Abstract

**Background:**

Veno-arterial extracorporeal membrane oxygenation (V-A ECMO) is critical for patients with severe cardiac or respiratory failure. Circuit damage, particularly at the proximal hard tube connector, can lead to complications such as blood leakage and hemodynamic instability. This case report examines the repair of a crack in the proximal hard tube connector during V-A ECMO treatment.

**Case presentation:**

A 70-year-old female receiving peripheral V-A ECMO support via femoral cannulation for acute heart failure suffered a crack in the proximal hard tube connector, causing arterial blood leakage. The team performed an emergency repair using bone wax while maintaining low-flow ECMO operation. The repair was successful, and V-A ECMO support was continued without interruption, with stable hemodynamics.

**Conclusion:**

Bone wax proved effective for repairing the V-A ECMO circuit crack in this case. The low-flow operation ensured uninterrupted V-A ECMO support and maintained hemodynamic stability, offering an alternative to full circuit replacement in similar cases.

## Introduction

Veno-arterial (V-A) extracorporeal membrane oxygenation (ECMO), most commonly established via peripheral femoral cannulation, is a life-saving treatment for patients experiencing severe cardiac or respiratory failure unresponsive to conventional therapies (1). By providing temporary cardiopulmonary support, ECMO supports systemic perfusion and gas exchange and may serve as a bridge to recovery or definitive therapy. However, prolonged ECMO use poses risks, mechanical failure in the circuit (2). Among circuit-related complications, tubing cracks or leaks are particularly critical in peripheral V-A ECMO, especially when they involve the patient-side arterial return cannula, especially the side-hole region near the distal cannula segment, which is designed to disperse high-pressure flow and protect downstream arteries. Failure at this site compromises both circuit integrity and vascular protection, leading to uncontrolled arterial bleeding, air embolism, and distal vascular injury. Unlike upstream components such as the oxygenator outlet, defects at the arterial cannula interface are difficult to isolate without interrupting V-A support, requiring immediate intervention (3). While circuit replacement is the standard approach, it requires interrupting ECMO support, which can risk critically ill patients safety (4). Finding a quick and effective temporary repair method could help avoid interruptions in treatment and reduce potential harm.

This study presents a case where a crack in the proximal hard-tube connector of a V-A ECMO circuit was successfully managed using bone wax. Bone wax is commonly used in surgery to stop bleeding, but in this case, it was used as a temporary sealant to prevent blood loss and air entry while keeping maintaining ECMO flow. This case report examines the repair of a crack in the proximal hard tube connector during V-A ECMO treatment.

## Case description

A 70-year-old female patient was admitted to the Fujian Provincial Hospital with chest pain and shortness of breath that had persisted for 10 days. The symptoms developed without an apparent cause, with the chest pain localized to the precordial region and described as a stabbing sensation. The patient reported orthopnea and mild abdominal discomfort, with no other symptoms or signs. She had a 10-year history of hypertension, with a recorded maximum blood pressure of 170/90 mmHg, and had been self-medicating, though the specific medications were unknown.

On admission, the patient had acute anterior myocardial infarction with markedly elevated cardiac biomarkers (high-sensitivity troponin I 3,195 ng/L, NT-proBNP 28,663 pg/mL). Bedside echocardiography demonstrated severe left ventricular dysfunction with segmental wall motion abnormalities and low stroke volume (VTI 7–9 cm). Clinically, she exhibited respiratory distress, hypoxemia, and high risk of hemodynamic collapse, consistent with cardiogenic shock. Chest CT revealed mild right lower-lobe exudation, pleural effusion, and cardiomegaly.

**Figure 1:**
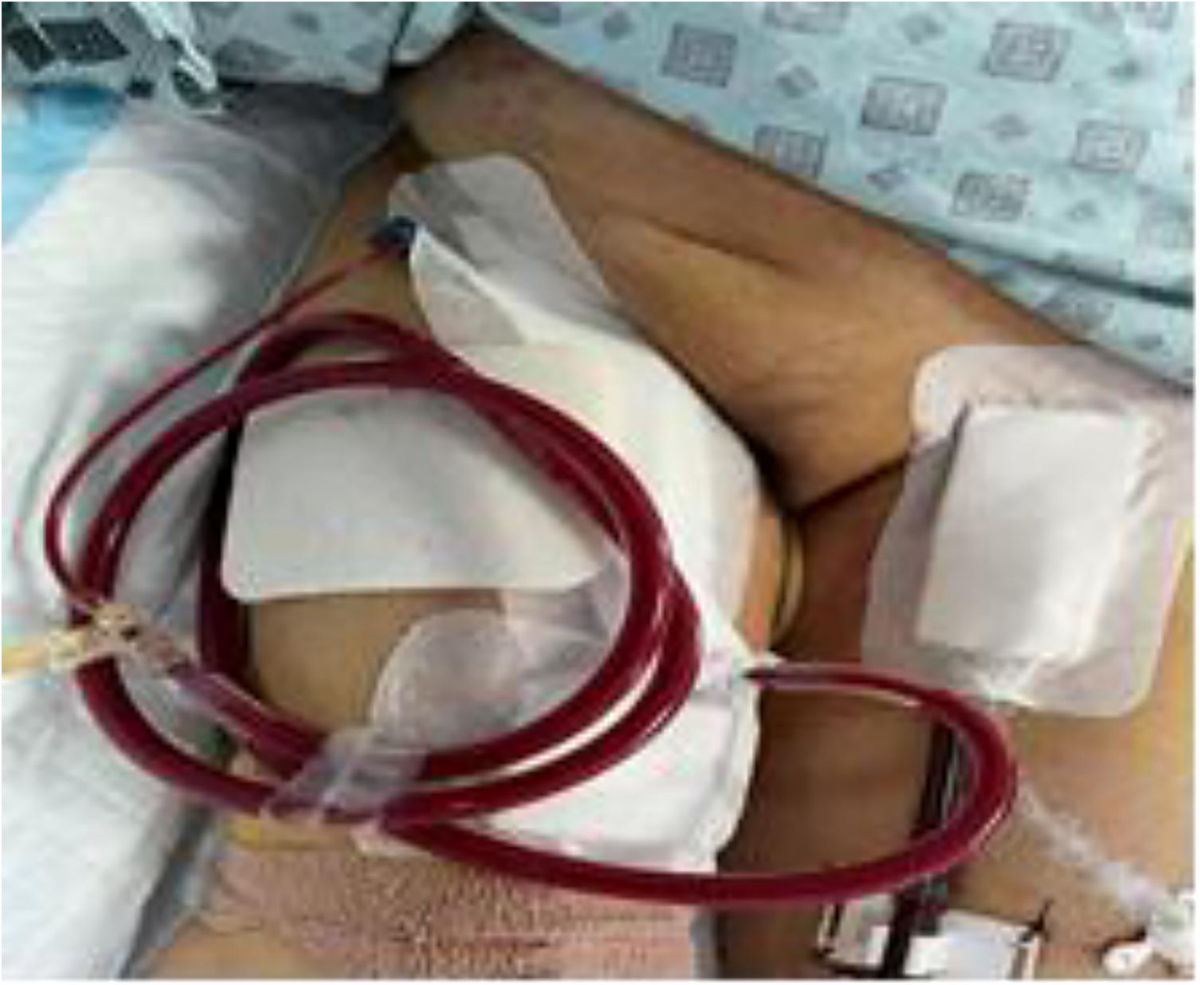
Patient underwent cannulation via the right femoral artery and left femoral vein.

The patient met the criteria for V-A ECMO (5) and underwent cannulation via the right femoral right femoral artery (arterial return) and left femoral vein (venous drainage) (Figure 1). Under ultrasound guidance, a 21F MAQUET ECMO arterial cannula was inserted 20 cm into the right femoral artery, while a 15F MAQUET ECMO venous cannula was inserted 44 cm into the left femoral vein, with intraoperative ultrasound confirming the catheter tip position at the right atrial inlet. Once the arterial and venous cannulas were connected to the ECMO circuit, the centrifugal pump was activated and gradually increased to 3,500 rpm, achieving a maximum flow rate of 3.0 L/min at 100% oxygen concentration. The patient's oxygen saturation (SpO₂) reached 100%, and arterial blood pressure (ABP) was recorded at 105/62 mmHg. The patient was supported with mechanical ventilation in SIMV PC + PS mode (FiO2 40%, PEEP 5 cmH2O, PC/PS 10 cmH2O, RR 10/min) throughout ECMO support. Oxygenation was primarily maintained via ECMO (FiO2 100%), with gas sweep flow adjusted to maintain arterial blood gases within target ranges. No evidence of differential hypoxemia (North-South syndrome) was observed.

At the 115th hour of ECMO operation, blood leakage was observed at the hard tube connection located 20 cm from the femoral artery cannula. Inspection revealed a crack (∼1.2 mm) at the side-hole region near the distal segment of the arterial return cannula (near the cannula tip), while the cannula itself remained intact. The ECMO circuit had been continuously monitored prior to the event, and no prior abnormalities were detected. Given the patient's critical condition, immediate circuit replacement was not feasible, and the event was classified as an emergency intervention. The ECMO team rapidly reduced flow to ∼1.0 L/min by lowering pump speed to 2,500 rpm, decreasing pressure at the crack site. No pre-optimization for low-flow support was performed, representing a limitation in this case. Bone wax (∼0.06 g) was applied directly to the cracked connector, and gauze strips were used to press the wax against the tube wall, facilitating absorption and sealing. This process was repeated until no further leakage occurred. The low-flow period lasted approximately 1 min, after which pump speed was gradually restored to 3,500 rpm, re-establishing a maximum flow rate of 3.0 L/min.

Throughout the 168-hour ECMO run, the arterial cannula site remained free of bleeding, and the repair remained intact with no further leakage. The patient was continuously monitored for potential embolization, including bedside x-ray assessment for bone wax emboli, and the ECMO cannulas were safely removed once hemodynamic stability was confirmed. Post-repair monitoring showed no signs of air embolism, thrombosis, or recurrent leakage. The patient tolerated the intervention well, and ECMO support was successfully weaned after 15 days. She remained hemodynamically stable throughout the repair process, and the emergency intervention prevented the need for circuit replacement, minimizing the risks associated with ECMO downtime. The patient was discharged spontaneously after decannulation without immediate complications. No follow-up data are available regarding long-term sequelae or morbidity related to ECMO cannulation.

## Discussion

This case report presented the repair of a crack in the proximal hard tube connector during V-A ECMO treatment, indicating that a combination of low-flow ECMO and bone wax application can be an effective emergency strategy for managing cracks in the ECMO circuit.

In this case, a sudden crack in the hard-tube connector caused active bleeding and posed an immediate threat. A similar concern was also reported by Yu and Zhou in a case involving ECMO support during a high-risk cardiac intervention, highlighting how even minor disruptions in ECMO function can lead to serious complications if not managed quickly (6). Stopping ECMO support entirely to fix a problem like this would typically be too risky. That's where the idea of “low flow” ECMO comes in. By reducing the pump speed and flow rate temporarily, pressure in the circuit drops, making it possible to perform emergency repairs while keeping support running. In our case, reducing the flow to 1.0 L/min helped control the leak long enough to carry out a safe and quick fix. Although Crawford et al. have reported the benefits of low-flow techniques during circuit repair in pediatric postcardiotomy ECMO (7), these findings may not be fully generalizable to adults with acute cardiogenic shock, whose hemodynamics and ventricular reserve differ substantially. Nevertheless, in this emergency scenario, the rapid, temporary low-flow approach provided the safest balance between maintaining circulatory support and allowing a timely repair, avoiding the potentially catastrophic consequences of full ECMO interruption. Systematic reviews and meta-analyses show that more than one-third of patients supported with V-A ECMO for cardiogenic shock experience serious complications, including bleeding, renal failure, and neurologic injury, and overall mortality rates remain high, highlighting the vulnerability of these patients when support is interrupted or compromised (8).

What made our case notable was the use of bone wax—a material with a long history of topical hemostatic use in cardiac surgical procedures, particularly for controlling bleeding from sternal bone edges after median sternotomy, rather than solely in orthopedic contexts. Bone wax mechanically occludes bleeding channels and provides immediate tamponade of bone surfaces and is familiar in thoracic surgery settings, although its use has been associated with impaired bone healing and sternal wound complications in some series, suggesting its application should be judicious even in surgical practice (9). A similar case was reported in 2003, in which bone wax was successfully applied to repair a cannula defect and maintain CPB function (10). There are also other emerging strategies for temporary ECMO circuit repair. For instance, Dermabond-like cyanoacrylate sealants have been reported for cannula leaks and circuit cracks, offering an alternative to bone wax in certain situations (11). However, these techniques carry potential risks. If excess bone wax is forced into the defect, it can enter the cannula lumen and embolize systemically, causing distal obstruction. Similarly, cyanoacrylate adhesives, if improperly applied, can generate embolic material or adhere to circuit components. Therefore, judicious application and careful monitoring are essential to minimize these hazards. In our case, bone wax was applied directly over the cracked connector and reinforced with gauze to achieve a seal, effectively stopping the leak and allowing uninterrupted ECMO support. Although we initially conceptualized this as a temporary sealant, in practice it functioned as the enduring solution for this particular patient, owing to the emergent context and her critical condition that precluded a planned circuit change.

While some reports have described reducing ECMO flow during circuit crises, it is important to note that this is primarily a procedural maneuver rather than a hemodynamic strategy. For example, Crawford et al. emphasize that flow reduction is used to prevent air entrainment during circuit troubleshooting, and their study specifies that definitive management should involve circuit change rather than prolonged low-flow support (7). Similarly, Kim et al. discuss rapid corrective measures during ECMO emergencies but do not advocate low-flow as a substitute for restoring full circuit integrity (12). In our case, brief low-flow operation (∼1 min) was employed solely to reduce pressure at the crack site and allow bone wax application, after which full flow was promptly restored. This distinction is important because prolonged low-flow in a patient with active cardiogenic shock could compromise end-organ perfusion and increase afterload, highlighting that this approach is only a temporizing measure in emergency repair, not a primary treatment strategy.

In this patient, cardiogenic shock was presumed to be ischemic in origin based on the presentation of acute anterior myocardial infarction, elevated cardiac biomarkers, and severe left ventricular dysfunction. At the time of haemodynamic deterioration, the patient was critically unstable with severe hypoxaemia and a high risk of circulatory collapse. Therefore, immediate coronary revascularisation, such as percutaneous coronary intervention (PCI) or coronary artery bypass grafting (CABG), was not performed. Instead, veno-arterial ECMO was initiated as a rescue therapy to rapidly restore systemic perfusion and oxygen delivery. Management priorities focused on haemodynamic stabilisation with intensive monitoring and anticoagulation guided by activated partial thromboplastin time. This strategy is consistent with current practice in refractory cardiogenic shock, in which VA-ECMO may serve as a bridge to recovery or to further therapeutic decision-making once physiological stability is achieved.

After the repair, we kept the patient under continuous monitoring to check for signs of air embolism, further bleeding, or circuit failure. Fortunately, the bone wax held up well over the 168 h ECMO run, with no signs of new leakage or other complications. The patient's hemodynamics remained stable, and we were eventually able to safely wean her off ECMO. This reinforces the value of proactive monitoring after any ECMO repair. Yu and Zhou also emphasize the need for vigilant monitoring after interventions, as this can help catch any issues early and improve the chances of a successful recovery (6). This study aligns with that, showing how timely repair, combined with close monitoring, can result in a good outcome even in high-risk cases.

Certain patient biometric parameters, including weight, height, and body surface area, were not available, which precluded calculation of BSA-indexed ECMO flow. Consequently, flow management relied on clinical monitoring and hemodynamic targets rather than standardized indexed values. This limitation may affect the generalizability of our findings and should be considered when applying similar emergency repair strategies. A limitation of this case is that the bone wax repair was performed out of necessity rather than as a standard permanent intervention. Ideally, ECMO circuit modifications are performed under controlled conditions with minimal flow interruption. In this patient, however, the risk of hemodynamic collapse precluded partial replacement, and the emergency repair served as the safest option to maintain continuous support.

This case shows that a combination of low-flow ECMO and bone wax application may be an effective emergency strategy for managing cracks in the ECMO circuit. It helped us avoid the risks of circuit replacement and maintain stable blood flow throughout the repair.

## Data Availability

The original contributions presented in the study are included in the article/Supplementary Material, further inquiries can be directed to the corresponding authors.
